# Differences in Managing Anticoagulants and Antiplatelets for Gastrointestinal Endoscopy between East and West

**DOI:** 10.4021/gr2009.04.1283

**Published:** 2009-03-20

**Authors:** Sun-Young Lee

**Affiliations:** Department of Internal Medicine, Konkuk University School of Medicine, 4-12 Hwayang-dong, Gwangjin-gu, Seoul 143-729, South Korea. E-mail: sunyoung@kuh.ac.kr

**Keywords:** Anticoagulation, Antiplatelet, Endoscopy, East, West

## Abstract

Decreasing the bleeding risk associated with gastrointestinal (GI) endoscopic procedures and minimizing the thromboembolic risk of withdrawing medications are very important for the patients taking anticoagulants and antiplatelets. Western guidelines on managing anticoagulation and antiplatelet medications in GI endoscopy suggest a polypectomy with aspirin medication or a biopsy with warfarin medication. However, Eastern endoscopists’ adherence to Western guidelines may be responsible for Easteners experiencing massive bleedings. During the cessation of drugs, it should be emphasized that Asians may be predisposed to different forms of embolism more likely to be of the cerebrovascular system, whereas Westerners more likely to be of the cardiovascular variety. To better understand the differences between the East and West, differences in drug metabolism should be considered that results in greater body weight-normalized plasma unbound clearance of drug in Easterners. Taken as a whole, different managements are required for GI endoscopy in patients on anticoagulation and/or antiplatelet medications based on differences in metabolism of drugs, risk of hemorrhage, and forms of thromboembolism.

## Introduction

Anticoagulants and antiplatelets may potentiate gastrointestinal (GI) bleeding due to medication, thromboembolism related to interruption of medication for endoscopic procedure, and bleeding after endoscopic procedure. A recent international survey has shown that there is a wide variation between the Eastern and Western endoscopists in managing anticoagulants and antiplatelets during the periendoscopic period [1]. This might be based on the differences in education, guidelines, genetics, environments, risk factors (diabetes mellitus, hypertension, hyperlipidemia, and obesity), prevalence of cardiovascular attack (stroke, deep vein thrombosis, and ischemic heart disease), effectiveness and side effects of antiplatelets or anticoagulants. For example, Eastern endoscopists frequently experience severe bleeding while following Western guidelines (ie, for a polypectomy with aspirin medication or a biopsy with warfarin medication) [2-5], and thus recent Eastern guideline recommends to withdraw either antiplatelets or anticoagulants even for a biopsy [6, 7]. Unfortunately, little is known about rationales on such differences between the East and West in managing antiplatelets or anticoagulants for GI endoscopy.

The aim of this review is to better understand the differences between the East and West by analyzing the: (1) East and West differences in endoscopists’ conception, (2) East and West differences in complications such as hemorrhage and embolism, and (3) East and West differences in drug metabolism.

## Differences in endoscopists’ conception between the East and West

Given the paucity of available data on different practice patterns between the Eastern and Western endoscopists, I and my colleagues performed an international survey to the GI endoscopists in Eastern (Korea, Japan, China, India, Thailand, Singapore, Malaysia, and Philippines) and Western (United States and Canada) countries [1]. It appeared that Eastern endoscopists do not typically perform an endoscopic biopsy while their patients are on warfarin therapy and do not perform a polypectomy while their patients are taking aspirin. In addition, there was a delay (1 to 3 days later) on restarting medications after an endoscopic biopsy or a polypectomy only in the Easternists. This occurred despite published Western guidelines that suggest it is safe to perform an endoscopic biopsy during warfarin therapy or a polypectomy while on aspirin medication [2-5], and despite a published paper stated that patients treated by the American Society of Gastrointestinal Endoscopy guidelines had 0% rate of thrombosis [8]. Through our survey [1], we could find that Eastern endoscopists believe it to be dangerous to follow Western guidelines because of an increased risk of bleeding in Asian patients. It seems that personal experience is a more powerful driver of practice than the published literature for the GI endoscopists.

### Management of drugs for diagnostic endoscopy

Most Eastern and Western endoscopists continued all medications when performing diagnostic endoscopy without a biopsy, and restarted it on the same day after the procedure [1]. After an endoscopic biopsy, the Eastern endoscopists tended to restart medications 1 to 3 days later, except for nonsteroidal anti-inflammatory drugs (NSAIDs) restarting on the same day, whereas the Western endoscopists restarted all medications on the same day. A recent European guideline makes the differences more definite by recommending warfarin and clopidogrel to be continued [5], while Japanese guidelines recommend cessation of aspirin and clopidogrel for low-risk procedures [6].

### Management of drugs for therapeutic endoscopy

Most Eastern and Western endoscopists withdrew all medications other than NSAIDs for a polypectomy, and restarted 1 to 3 days after a polypectomy [1]. Main difference between the East and West was found on managing aspirin for a polypectomy. Eastern endoscopists tended to withdraw aspirin for more than 7 days before a polypectomy, whereas Western endoscopists performed a polypectomy without withdrawing aspirin medication. In addition, Eastern endoscopists restarted aspirin 1 to 3 days after a polypectomy, whereas Western endoscopists restarting it on the same day [1].

### Publications on managing antiplatelets and/or antiplatelets for endoscopy

Great differences between the East and West were found in published articles on managing anticoagulants and antiplatelets for GI endoscopy (Table 1) [1, 2, 4-6, 8-15]. To explain this discrepancy, differences in effectiveness and side effects of antiplatelets and anticoagulants between the East and West should be discussed with the differences in complications such as bleeding and thromboembolism [16]. Although it is difficult to conclude the superiority, it should be reminded that life threatening bleeding is rare when compared to the risk of thromboembolism. Bleeding usually result in less morbidity and can be managed by the endoscopists, whereas major thromboembolic events during discontinuation of drugs often lead to death or permanent disability [7, 8, 17]. Cultural difference should be also considered for such difference because it might be speculated that Western gastroenterologists would limit anticoagulant abstinence for their patients, because of more litigious nature of medicine in Western societies.

**Table 1:** Published papers on managing antiplatelets and/or anticoagulants for GI endoscopy (in recent order)

Country (author, year)	Key Messages
Japan (Fujishiro M, 2009) [8]	• Aspirin, ticlopidine, and ethyl icosapentate were stopped 7 days before the procedures by most of the endoscopists, whereas warfarin was stopped 4 days before the procedures. • With regard to the drug discontinuation before the procedures, no major differences were observed between a biopsy (low-risk procedure) and endoscopic mucosal resection (high-risk procedure). • In case of higher risk of thromboembolism during the cessation of drugs, most of the endoscopists never performed a biopsy or endoscopic mucosal resection.
Korea (Lee SY, 2008) [1]	• Eastern endoscopists do not typically perform an endoscopic biopsy while their patients are on warfarin therapy. • Eastern endoscopists restarted medications later (1 to 3 days) than Western endoscopists after a biopsy (same day). • Eastern endoscopists do not perform a polypectomy while their patients are taking aspirin. They withdrew aspirin for more than 7 days before a polypectomy, and restarted it 1 to 3 days after a polypectomy.
UK (Veitch A, 2008) [5]	• Low-risk procedures can be performed during warfarin or clopidogrel intake. • High-risk procedures should be done after stopping warfarin for 5 days (INR < 1.5), and substituted with LMWH in high-risk conditions. • In low-risk conditions, high-risk procedures can be performed after stopping clopidogrel for 7 days. Aspirin should be considered during the cessation of clopidogrel in such conditions. • In high-risk conditions, continuing aspirin and stopping clopidogrel for 7 days should be considered, if 12 months have passed drug-eluting coronary stent insertion.
UK (Goel A, 2007) [10]	• With regard to warfarin, 26% of the endoscopists stopped for EGD, whereas 48.7% stopped for colonoscopy. • During warfarin intake, 21% took biopsies as usual, while 54% performed after checking the INR. • Warfarin was usually stopped for 3 days. Preferred INR was below 2.0 during the endoscopic procedure.
Israel (Kimchi NA, 2007) [11]	• Aspirin can be continued for diagnostic EGD, but should be stopped 5 to 7 days before colonoscopy, spincterotomy, esophageal dilation, endoscopic ultrasound-guided biopsy, or drainage. • Aspirin use should be avoided for 2 weeks after polypectomy, and 10 days after spincterotomy. However, when the cardiovascular risk is high, aspirin should be resumed within a week after the procedure. • NSAIDs should be stopped 8 hours before any endoscopy, and be resumed 7 to 14 days after a high-risk procedure. • Clopidogrel should be stopped 5 days before colonoscopy.
US (Makar GA, 2006) [12]	• For low-risk procedure (e.g. diagnostic endoscopy and colonoscopy without polypectomy), there is no need to discontinue or adjust anticoagulation. • For high-risk procedures (e.g. polypectomy and biliary sphincterotomy), an individual approach is required. This approach includes stopping oral anticoagulant therapy with or without the administration of unfractionated heparin or LMWH during which the patient’s INR is in the subtherapeutic range. • Antiplatelet therapy can be withheld for high risk procedures, but there is insufficient evidence to indicate that bleeding risk is impacted.
France (Naploen B, 2006) [4]	• Low-risk procedures can be performed during medications. • High-risk procedures and transnasal endoscopy should be performed after the cessation of drugs. • Colon polypectomy and endoscopic sphincterotomy could be performed without cessation of aspirin or NSAIDs.
Japan (Ogoshi K, 2005) [6]	• Warfarin should be stopped 3 to 4 days before all kinds of endoscopic procedure. INR less than 1.5 is preferred during the procedure. • Aspirin should be stopped 3 days before low-risk procedures, and stopped 7 days before high-risk procedures. • Ticlopidine should be stopped 5 days before low-risk procedures, and stop 10 to 14 days before high-risk procedures. • In case of dual antiplatelet intake (aspirin and ticlopidine), both antiplatelets should be stopped 7 days before low-risk procedures.
Germany (Mosler P, 2004) [13]	• Antiplatelets were usually continued before elective procedures including diagnostic EGD, colonoscopy, and ERCP. • Aspirin and clopidogrel were more frequently stopped than NSAIDs prior to any endoscopic procedure. • More than 80% stopped aspirin and clopidogrel before elective therapeutic endoscopic procedures. • Warfarin was usually discontinued 3-5 days prior to the procedure, and was substituted with LMWH.
Belgium (Hittelet A, 2003) [14]	• It is not necessary to discontinue aspirin or NSAIDs for endoscopic procedures, when used in standard doses. • It is not necessary to adjust anticoagulation for low-risk procedures, such as EGD, colonoscopy, ERCP with biopsy or stent insertion (without sphincterotomy). • For ticlopidine or clopidogrel, it is prudent to discontinue 7 to 10 days for high-risk procedures. • Warfarin should be discontinued 3 to 5 days before high-risk procedures.
US (Eisen G, 2002) [2]	• Any endoscopic procedure may be performed in patients taking aspirin or NSAIDs in the absence of pre-existing bleeding disorders. • Discontinuation of oral anticoagulation is needed for high-risk endoscopic procedures. • Consider a heparin window only for patients with high thromboembolic risk. • Resumption of warfarin is generally recommended at the night of the procedure except for sphincterotomy.
US (Kadakia SC, 1996) [15]	• Physicians stopped aspirin and NSAIDs more frequently before colonoscopy and ERCP than before EGD. • With aspirin or NSAIDs intake, cold biopsy or hot biopsy during EGD or colonoscopy was performed, but sphincterotomy was not. • Optimal cessation period of aspirin or NSAIDs was less than 10 days. • Warfarin was resumed immediately after diagnostic endoscopy, whereas 7 days of cessation period was observed after therapeutic endoscopy.

Note: INR, international normalized ratio; LMWH, low molecular weight heparin; EGD, esophagogastroduodenoscopy; ERCP, endoscopic retrograde cholangiopancreaticography; NSAIDs, nonsteroidal anti-inflammatory drugs.

Taken as a whole, racial differences in susceptibility to complications have led to establish different guidelines between countries, especially between the East and West. A large, prospective, randomized, and double-blinded clinical study might be a solution, but it is very dangerous and unethical to perform such a prospective study in patients on antiplatelets or anticoagulants. Therefore, opinion of the experts or review papers should be an alternative proposal.

## Differences in complications between the Easterners and Westerners

Complications in patients on anticoagulation and/or antiplatelet medications can be divided into three categories: (1) spontaneous GI bleeding from medication; (2) GI bleeding related to an endoscopic procedure; and (3) thromboembolism from cessation of medications.

### Embolism

High risks for thromboembolisms are valvular heart disease with atrial fibrillation, mechanical valve with past history of thromboembolism, and mechanical valve in mitral area [2]. Of these risk factors, atrial fibrillation is most dangerous factor that increases thromboembolic risk with older age more than 80 year-old, past or family history of cerebrovascular embolism, hypertension, congestive heart failure, diabetes mellitus, or hyperlipidemia [18].

In Western studies, it has been reported that acute coronary syndrome is not rare within one month of aspirin withdrawal [19, 20]. In contrary, it seems that Asians are more susceptible to cerebral stroke than Caucasians, whereas Caucasians are more susceptible to other cardiovascular attacks including deep vein thrombosis (Table 2) [7, 8, 17, 21-23]. Therefore, it should be emphasized again that thromboembolism affecting Westerners may be more likely to be of the cardiovascular variety, while that of Easterners may be more likely to be of the cerebrovascular variety. It should be stressed again to Eastern endoscopists, because cerebral infarction during discontinuation of the anticoagulants or antiplatelets often leads to permanent disability or death as observed in previous Eastern reports [7, 8, 17].

**Table 2:** Reports on embolism that happened during the cessation of antiplatelets and/or anticoagulants

	Country (author, year)	Frequency	Type of embolism
**East**	Korea (Lee SY, 2006) [17]	Six of 81 (7.4%) endoscopists have experienced embolism during past one year.	5 cerebral infarction 1 mesenteric infarction
	Japan (Ishizawa T, 2006) [7]	Seven of 81 (8.6%) endoscopists have experienced embolism during past three years.	5 cerebral infarction 2 myocardial infarction
	Japan (Fujishiro M, 2009) [8]	Three of 13 (23.1%) endoscopists have experienced embolism during their career.	2 cerebral infarction 1 mesenteric infarction
**West**	US (Dunn A, 2003) [21]	Twenty nine of 1868 (1.6%) patients undergoing dental or orthopedic procedures, or cataract surgery have experienced thromboembolic events.	21 cardiovascular embolism including peripheral arterial thromboembolism 7 cerebral infarction 1 unspecified
	US (Garcia D, 2008) [22]	Seven of 1024 (0.7%) patients showed embolism during the study period.	3 cerebral infarction 2 pulmonary embolism 1 deep vein thrombosis 1 mesenteric infarction
	US (Kuwada SK, 1996) [23]	One of 27 (3.7%) patients showed embolism during the study period.	1 peripheral arterial thromboembolism

According to recent data on death rates from ischemic heart disease and stroke among nations, the incidence of stroke in Korea and Japan is higher than that of ischemic heart disease [24], despite rapid increasing rate of cardiovascular disease in Asian countries since 1985 [25]. However, the rise in risk factors due to westernized life style in Japan did not result in an increase in coronary heart disease mortality and morbidity like in US, even in the post-World War II cohorts [25]. The ratio of ischemic heart disease to stroke is highly different between the East and West, although the prevalence of thromboembolism and their subtypes are getting similar throughout the world [24]. Therefore, we can speculate that genetic factor might be involved in addition to environmental factor such as westernized dietary habits (higher fat intake, lower polyunsaturated/saturated ration, and less omega-3 fatty acids). Such an ethic difference suggests that there might be some powerful and important genetic factors in Asians, and identification of such factors would clearly have great importance for prevention of cardiovascular diseases.

### Bleeding

Endoscopy-induced bleeding can classified into high-risk procedures (bleeding rate > 1%) and low-risk procedures (bleeding rate < 1%) [2]. High-risk bleeding procedures include tissue resections such as polypectomy, mucosectomy, anpullectomy, endoscopic submucosal dissection, sphincterotomy, bougiennation, pneumatic or balloon dilation, metal stent insertion, percutaneous endoscopic gastrostomy, endoscopic ultrasonograph guided fine needle aspiration, laser ablation, photocoagulation, hemostatic procedures, and variceal ligation. According to the Western guidelines [2-5], these procedures require cessation of drug, while low-risk procedures do not. Notably, transnasal esophagogastroduodenoscopy (EGD) should be managed as high-risk procedures because of epistaxis, whereas biliary or pancreatic stenting should be managed as low-risk procedures because of their relative safety [4].

In contrast to the outcomes of thromboembolic events from withdrawing antiplatelets and anticoagulants, hemorrhagic complications from endoscopic procedures are less likely to result in death or permanent disability. However, despite all these publications from the West, the risk of procedure-related bleeding is still heavily weighted toward the risk of a thromboembolism in the East [1]. For example, Eastern endoscopists do not follow Western guidelines (ie, for a polypectomy with aspirin medication or a biopsy with warfarin medication) because it may be responsible for patients experiencing massive bleeding [1, 6-8, 17].

Anticoagulants are not by themselves ulcerogenic, but associated with a greater risk of upper GI bleeding because of an exacerbation of pre-existing lesions in the GI tract associated with NSAIDs, aspirin, or*Helicobacter pylori* infection [26]. Predictors of bleeding include past history of GI bleeding or ulcer disease, higher intensity of anticoagulation, old age more than 65 year-old, combination therapy (anticoagulants with antiplatelets), and presence of comorbid conditions like chronic renal failure, congestive heart failure, diabetes mellitus, or alcoholic liver disease [27]. Highest bleeding risk happens with warfarin followed by aspirin, NSAIDs, ticlopidine, clopidogrel, dipyridamole), and cyclooxygenase-2 (COX-2) inhibitor [28]. Of endoscopic findings, peptic ulcer is the most common, followed by hemorrhagic gastritis, and esophagitis [29].

In case of GI bleeding, complete or partial reversal of anticoagulation is undertaken based on the balance of risks between bleeding and thromboembolism. Early endoscopy can reveal lesions requiring endoscopic hemostasis, which can be performed in the setting of low-intensity anticoagulation [29, 30]. For the hemostasis of GI bleeding during warfarin intake, partial reversal of anticoagulation to international normalized ratio (INR) 1.5 to 2.5 with fresh frozen plasma can allow for successful endoscopic hemostatic therapy [30]. Fresh frozen plasma should be used instead of vitamin K, because vitamin K infusion often leads to thromboembolic complication after an urgent infusion [23]. Thrombin spraying or hemoclipping could also be considered with dried human blood coagulant IX factor complex 500 unit to normalize the prolongated INR [31]. Platelet concentrate transfusion should be considered in case of antiplatelet intake, and protamine sulfate injection should be considered in case of heparin infusion.

Decision for discontinuation of drugs in the setting of GI bleeding must be made on an individual basis, based upon potential thrombotic and hemorrhagic risks. It seems prudent to briefly discontinue the drugs until lack of rebleeding, because hemodynamic instability and hemostatic changes induced by acute GI bleeding may further increase the risk of thrombosis without medications [32]. In case of longer period of cessation, low molecular weighted heparin (LMWH) should be considered instead of warfarin [33]. When it is difficult to stop aspirin, proton pump inhibitor (PPI), misoprostol, or COX-2 inhibitor should be considered [34]. In patients with a past history of peptic disease or bleeding from an acid-related lesion, PPI and *H. pylori* eradication should be considered to reduce the risk of upper GI bleeding even with antiplatelet intake [32, 35].

## Differences in drug metabolism between the Easterners and Westerners

Medications that may potentiate GI bleeding has become more widespread these days (Table 3). These drugs are classified into (1) antiplatelets such as glycoprotein IIb/IIIa inhibitors, adenosine diphosphate receptor antagonist, prostaglandin analogue, COX inhibitors, etc; (2) anticoagulants such as vitamin K antagonists, direct thrombin II inhibitors, direct factor Xa inhibitors, heparin groups, glycosaminoglycans, etc; (3) thrombolytic drugs/fibrinolytics; and (4) others such as non-medicinal.

**Table 3:** Medications that may potentiate GI bleeding (in alphabetical order)

Drug	Half life	Time of action	Mechanism of action
Abciximab	0.7 hours (in alpha), 10 hours (in beta)	0.5-2.5 hours	Nonspecific antagonist for glycoprotein IIb/IIIa receptor.
Anagrelide hydrochloride	76 hours	Long	Reduction in platelet production resulting from a decrease in megakaryocyte.
Aspirin	0.25-19 hours (depends on dose)	0.5-5 hours	Irreversibly acetylates and inactivates cyclooxygenase, and thereby inhibits platelet production of thromboxane A2.
Beraprost sodium	1 hour	0.5 hour	Reversibly exacerbates adenylcyclase activation (reversible within 8 hours).
Clopidogrel	7-8 hours	2 hours	Same with ticlopidine, but has less side effects such as severe neutropenia and thrombotic thrombocytopenic purpura than ticlopidine.
Cilostazol	11-13 hours	3-6 hours	Inhibition of phosphoestrase (reversible within 48 hours).
Dilazep dihydrochloride	4 hours	0.5-1 hour	Reversibly inhibits phosphoestrase.
Dipyridamole	1.7 hours	2-3 hours	Reversibly inhibits phosphoestrase and inhibits uptake of adenosine.
Ethyl icosapentate	< 24 hours	Long	Irreversibly inhibits thromboxane A2 production.
Heparin	0.5-2.5 hours	Immediately	Activates antithrombin III, accerelates the rate of inhibiting clotting enzymes, particulary thrombin and factor Xa.
Ifenprodil tartate	1.4 hour	Short	Inhibits binding to serotonin 5HT2 receptor.
Nonsteroidal anti-inflammatory agents	< 24 hours	0.5 hours	Reversibly inhibit platelet cycloxygenase.
Ozagrel sodium	1.5 hour	Short	Inhibits enzymatic synthesis of thromboxane.
Sarpogrelate hydrochloride	0.7 hour	1.5 hour	Reversibly inhibits binding to serotonin 5HT2 receptor as a selective antagonist.
Ticlopidine	12.6 hours	6 hours	Irreversibly inhibits the binding of adenosine diphosphate to platelet cell-surface adenosine diphosphate (P2) receptor, and the subsequent ADP-mediated activation of the glycoprotein IIb/IIIa receptor.
Tirofiban	1.5-3 hours	0.1 hour	Specific antagonist for glycoprotein IIb/IIIa receptor.
Trapidil	2-4 hours	0.5-2 hours	Reversibly inhibits phosphoestrase, reversibly inhibits thromboxane A2 production.
Triflusal	0.5 hour	24 hours	Inhibits platelet arachidonic acid metabolism.
Warfarin	36-42 hours	72-96 hours	Prohibit the synthesis of Vitamin K dependent coagulation factor (II, VII, IX, X) in the liver Vitamin K is used as an antagonist.

Informations obtained at http://www.rxlist.com, http://kimsonline.co.kr, and http://www.druginfo.co.kr.

### Warfarin

**Figure 1:**
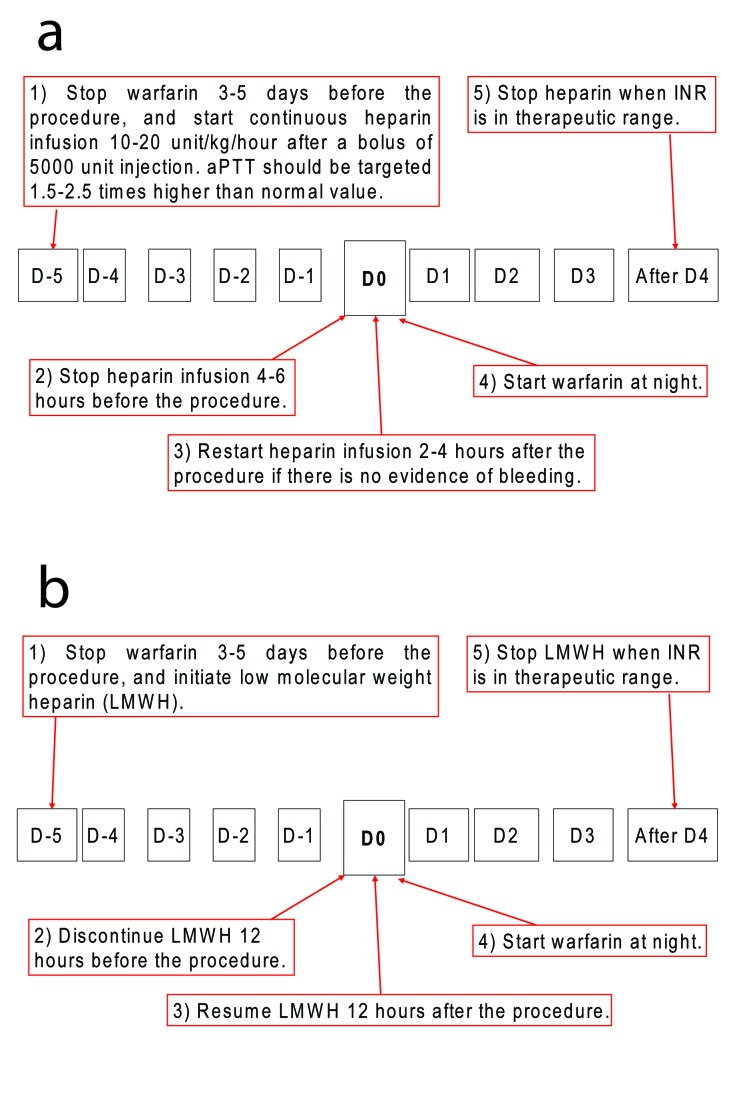
Recommendations on managing anticoagulants for high-risk patients undergoing high-risk procedures. (a) Warfarin substituted with intravenous heparin infusion. Warfarin should be stopped 3-5 days before the procedure, and be substituted with unfractionized heparin during the cessation period. (b) Warfarin substituted with subcutaneous heparin injection. Subcutaneous low molecular weighted heparin (LMWH) can be used instead of unfractionized heparin. Risk of thromboembolic complications must be carefully weighed against the increased risk of bleeding by maintaining anticoagulation.

A wide variation exists in managing warfarin before an endoscopic procedure, since the management of perioperative anticoagulation therapy for patients having a high risk of thromboembolism who are receiving long-term oral anticoagulant therapy is still uncertain. The prevalent approach is to discontinue oral anticoagulation therapy and initiate heparin therapy (Figure 1) [36]. Another potential strategy is to continue oral anticoagulation therapy with a temporary adjustment of warfarin intensity to a preprocedural INR. For example, a Western study showed a very low risk of bleeding when clips are applied immediately after polypectomy in anticoagulated patients at high risk of thrombosis [37]. It should be emphasized that recommended INR in the West is below 2.0 [2, 37, 38], while that of the East is below 1.5 for an endoscopic procedure [6].

An appropriate prothrombin time-INR seems to be lower in the Easternist than in the Westernist. INR values between 1.6 and 2.6 seem optimal to prevent ischemic or haemorrhagic events in Japanese, because bleeding complications during warfarin medication seems to be higher in Japanese [39]. An INR of 2.0 to 3.0 in Caucasians is equal to an INR of 1.6 to 2.8 in Japanese when checked by the thrombo test. It has been also reported that lower intensity warfarin (INR 1.5 to 2.1) treatment may be safer for the secondary prevention of stroke in Japanese patients with nonvalvular atrial fibrillation with presumed cardioembolic transient ischemic attack or stroke within the previous 6 months, than conventional-intensity (INR 2.2 to 3.5) warfarin treatment, because major haemorrhagic complications are avoided [40]. In this multicenter, prospective, randomized trial, optimal intensity of warfarin therapy for secondary prevention of stroke in Japanese patients with nonvalvular atrial fibrillation was INR 1.5 to 2.1, showing a lesser major hemorrhagic complication than conventional INR 2.2 to 3.5 without difference in thromboembolic complication.

Such differences seem to come from the difference in warfarin metabolism between the Easterners and Westerners. Japanese patients receiving warfarin therapy had a significantly greater body weight-normalised plasma unbound clearance of S-warfarin than white patients [41, 42]. Caucasian and Japanese patients who carried CYP2C9 variants possessed a lower unbound oral clearance for S-warfarin (decreased metabolic activity), thereby required a smaller daily dose of the drug [42]. In addition, Japanese possessing the wild-type promoter and coding sequences had significantly greater CYP2C9 activity than white patients with the corresponding genotype [43]. Recently, a pharmacological dose algorithm for warfarin that uses genotypes from two genes (VKORC1 and CYP2C9) and clinical variables was developed to predict the stable therapeutic dose [42]. Those carrying the VKORC1 1173C/C wild-type allele needed higher unbound concentrations of S-warfarin to achieve a therapeutic anticoagulation response (reduced sensitivity), and a greater daily dose was required [42]. Apart from the genetic variability in determining the dose of warfarin, diet might play a role in bleeding time and in response to warfarin. Patients with high fish diets eat a lot of omega-3 fatty acid which inhibits thromboxane production. In addition, patients with low vitamin K level would require smaller dose of warfarin. Recently, an algorithm was developed for estimating the appropriate warfarin dose that is based on both clinical and genetic data from a broad population base [44].

### Heparin

Heparin should be stopped 4 to 6 hours before the procedure and restarted 2 to 6 hours later after the procedure if there is no evidence of bleeding (Figure 1) [36]. LMWH consisted of smaller fragments of heparin, has replaced heparin because of its advantages such as a better bioavailability and longer half-life after subcutaneous injection, dose-independent clearance, predictable anticoagulant response, lower risk of heparin-induced thrombocytopenia, and osteoporosis [45]. In addition, LMWH has been demonstrated to cause less bleeding that unfractionized heparin because it binds to platelets, and thus is less likely to interfere with the interaction between platelets and the vessel wall [45]. Moreover, an advantage of LMWH over unfractionated heparin is that perioperative conversion from warfarin therapy with LMWH can be carried out in the outpatient. However, lack of a readily available laboratory test for monitoring is a great disadvantage, since endoscopists cannot be certain of the amount of anticoagulant effect present at the time of procedure. Since antifactor Xa activity may persist up to 12 hours after a single subcutaneous injection, the best test to monitor activity is an antifactor Xa assay, but this takes a long process time [3, 45]. LMWH is only partially reversible by protamine sulfate, and there is no proven evidence in the safety and effect for GI endoscopy [46, 47].

### Aspirin

There are Western reports showing that the administration of aspirin at standard dosages does not significantly increase the risk of bleeding after an endoscopic biopsy, colon polypectomy, or endoscopic sphincterotomy [48-52]. However, aspirin is known to cause a significant GI bleeding after endoscopic procedures in the Easternists, and thus recommended to stop not only for polypectomy but even for a biopsy [6-8]. A retrospective study from Honkong is the only Asian study that encouraged polypectomy without cessation of aspirin [50]. Of their 1657 subjects who underwent colon polypectomies, 127 subjects were taking aspirin, and 4.72% (6/127) revealed post-polypectomy bleeding. Notably, another study from Hongkong showed that aspirin therapy increased the risk of postsphincterotomy bleeding [53]. They reported that withholding aspirin for one week before endoscopic sphincterotomy did not seem to decrease the risk of post-sphincterotomy bleeding, because aspirin induces a long-lasting functional defect in platelets. These opposite results from Hongkong made problem unsolved, because the risk of postpolypectomy bleeding (1.4%) is known to be identical to that of postpolypectomy bleeding (1.4%) [54, 55]. Therefore, it is not strange that most of the Eastern endoscopists stop aspirin for a polypectomy [1].

Actual effects of aspirin are likely to differ by race and ethnicity. Although Western reports showed that aspirin does not affect GI bleeding time [48], significant prolongation was noted in colonic bleeding time after aspirin ingestion in Easternists [56]. Indeed, the threshold of antiplatelet therapy for Asian populations is considered to be 2 to 5 times higher than those for the US population, because of higher risks of hemorrhagic complications [57]. In addition, it has been reported that prevalences of gastrodeuodenal mucosal injury and bleeding are higher in Japanese receiving antiplatelet agents [58]. The higher estimated incidence of major GI bleeding might be related to the higher prevalence of *H. pylori* infection in this population. In a study case-control study of 695 consecutive users of low-dose aspirin with upper GI bleeding, *H. pylori* infection was identified as an independent risk factor of upper GI bleeding [59]. Besides, prophylaxis with a PPI effectively prevents recurrent upper GI bleeding with low-dose aspirin, despite failure of *H. pylori* eradication and concomitant use of NSAIDs [32]. Taken altogether, recommended dose of aspirin should be smaller in the Easternists because low-dose aspirin (75-150 mg) is effective, and because high-dose aspirin (> 100-200 mg) produces double rate of bleeding compared with low-dose aspirin (75-100 mg) per day [24, 57].

Notably, there were few Western studies that showed bleeding risks during aspirin intake. Minor bleeding occurred after biopsy or polypectomy at EGD or colonoscopy in 20 of 320 (6.3%) patients who had recently consumed aspirin or NSAIDs compared with 8 of 374 (2.1%) patients who had not [49]. Even in low-dose aspirin, major right sided colonic hemorrhage occurred after hot biopsy of diminutive colonic polyps [60]. Among elderly patients, the odd ratios of bleeding with daily doses of aspirin of 75, 150, and 300 mg were 2.3, 3.2, and 3.9, respectively [61]. In this study, the use of low-dose aspirin was associated with a two to fourfold greater risk of upper GI event, which is not reduced by the use of buffered or enteric-coated preparations [32, 61].

### Non-aspirin NSAIDs

Both the Eastern and Western endoscopists do not stop NSAIDs for endoscopic procedure [1], NSAIDs does not appear to prolong mucosal bleeding time [62], and does not increase the incidence of major bleeding [49]. Among non-aspirin-NSAIDs, aceclofenac had the lowest risk of upper GI bleeding, whereas ketorolac had the highest [63]. In this study, rofecoxib increased the risk of upper GI bleeding, whereas celecoxib, paracetamol or concomitant use of a PPI with an NSAID presented no increased risk. However, when combined with low-dose aspirin, the differences between non-selective NSAIDs and coxibs tend to disappear [63]. An apparent interaction was found between low-dose aspirin and use of non-aspirin-NSAIDs, coxibs or thienopyridines, which increased risk of upper GI bleeding. Treatment with either non-aspirin antiplatelet or cardioprotective aspirin has a similar risk of upper GI bleeding [32, 63].

### Platelet cell-surface adenosine diphosphate receptor antagonist (ticlopidine, clopidogrel, dipyridamole)

Platelet cell-surface adenosine diphosphate receptor antagonist includes thinopyridines (ticlopidine and clopidogrel) and dipyridamole. Ticlopidine and clopidogrel are recommended to be ceased 7 to 10 days before the procedure [3, 14]. In a recent European publication, clopidogrel is recommended to be ceased only for the high-risk procedures [5]. Besides, Japanese studies recommends 3 days of cessation period for aspirin, 5 days for ticlopidine, and 7 days for dual aspirin and ticlopidine therapy even for a biopsy [6, 64, 65]. Notably, dipyridamole does not increase bleeding risk, even with a dual antiplatelet therapy with aspirin [66].

Ticlopidine are more likely to induce endoscopic evidence of mucosal damage than patients taking aspirin or NSAIDs [28]. Besides, substitution of clopidogrel for aspirin is not recommended to reduce the risk of recurrent ulcer bleeding in high-risk patients, because their anti-angiogenic effects may impair healing of gastric erosions or small ulcerations that develop from other medications or *H. pylori* infection [32]. In summary, to minimize GI bleeding, history of ulcer and other GI risk factors should be assessed first, followed by test for *H. pylori *and treat if infected. PPI reduces the bleeding risk in most cases [32, 63].

### Glycoprotein IIb/IIIa receptor antagonist (etifibatide, abciximab, tirofiban)

Glycoprotein IIb/IIIa receptor antagonists are intravenously administered drugs given as a bolus followed by a continous infusion in acute coronary syndrome. There is no guideline for glycoprotein IIb/IIIa receptor antagonists, and it seems that a considerable number of endoscopists are unaware of these drugs [1]. Platelet functions are known to be recovered within 4 hours after the cessation of tirofiban and eptifibatide, whereas 24 hours are required for abciximab [12]. The antiplatelet effects can be partially reversed by platelet infusions or desmopressin (DDAVP) [3].

### Collaboration between the endoscopist and physicians prescribing drugs

Collaboration between the endoscopists and other engaged physicians (cardiologists, neurologists, surgeons, or general practitioners) are important to balance the risks of bleeding and thrombosis when deciding the cessation of antiplatelets and/or anticoagulants. Such collaboration will help endoscopists to make decision more appropriately by minimizing cardiovascular concerns. However, it seems that only 61.9% of Eastern endoscopists and 56.0% of Western endoscopists usually consult with the referring physician who prescribed the medications [1]. Reasons why many endoscopists do not consult primary care physicians about management of antiplatelet and/or anticoagulation medications might be related to (1) patient factors; (2) endoscopist factor; and (3) external factor [67]. First, some people take aspirin simply for cardioprotective reasons without significant disease, and thus endoscopists do not consult primary physicians in such situations. Second, endoscopists usually do not perform high-risk endoscopic procedures on patients taking warfarin except in emergency situations. That is, endoscopists usually defer elective procedures in high-risk patients. Third, there is no appropriate guideline that covers the exact situation in each patient. Personal experience seems to be more powerful driver of practice than published literature as noted in our survey [*1*].

**Figure 2:**
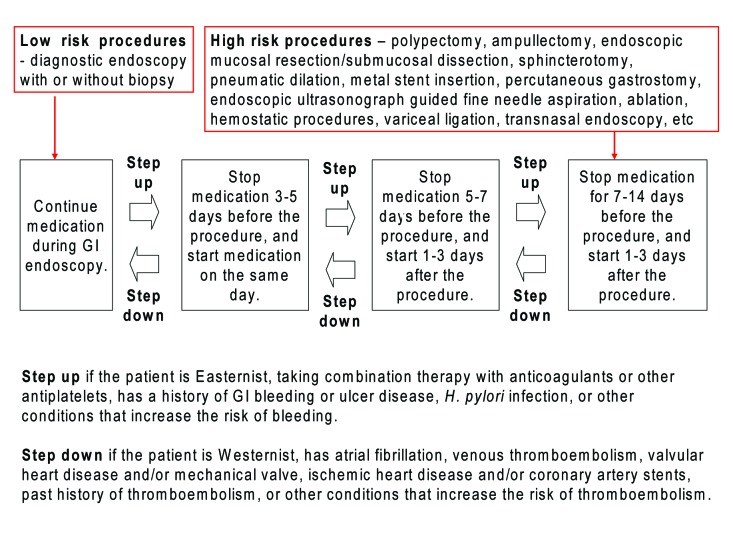
Recommendation on managing antiplatelets for GI endoscopy by risk stratification. For low-risk procedures, GI endoscopy could be performed without discontinuing the antiplatelets. If the patient is Easternist, taking dual therapy with anticoagulants or other antiplatelets, has a history of GI bleeding or ulcer disease, H. pylori infection, or other conditions that increase the risk of bleeding, cessation of drugs should be considered (marked as “Step up” with an arrow in the schema). For high-risk procedures with more than 1% of bleeding complication rate, cessation of antiplatelets should be considered at least 1 week before the procedure, and restarted when there is no evidence of bleeding. If the patient is Westernist, has atrial fibrillation, venous thromboembolism, valvular heart disease, mechanical valve, ischemic heart disease, coronary artery stents, past history of thromboembolism, or other conditions that increase the risk of thromboembolism, shorter cessation of antiplatelets should be considered (marked as “Step down” with an arrow in the schema). The degree of stepping up and down should be decided according to the number and severity of risk factors written above.

Not knowing the importance of collaboration and measuring the risks/benefits in each individual patient on a case-by-case basis, it would be very difficult to estimate the risks. A schema weighting the risk of bleeding and thromboembolism might be suggested (Figure 2) considering the differences between the East and West (Table 4).

**Table 4:** Summary on major differences between the East and West

	East	West
Risk of embolism	Lower than the Westerners. Common form is cerebrovascular variety that may lead to death or disability.	Higher than the Easterners. Common form is cardiovascular variety including deep vein thrombosis.
Risk of bleeding	Higher than the Westeners due to different drug metabolism (greater body weight-normalized plasma unbound clearance of drug) and higher rate of *H. pylori* infection.	Lower than the Easterners. Tolerates well with low-risk endoscopic procedures during antiplatelet and/or anticoagulant medications.
Managing warfarin	Lower international normalized ratio value (1.6-2.6) than the Westerners are appropriate for prophylaxis of thromboembolism.	Tolerates well with low-risk procedures (endoscopic biopsy) without significant bleeding.
Managing aspirin	Lower dose is recommended than the Westerners due to higher risk of bleeding.	Tolerates well with few high-risk procedures (endoscopic sphincterotomy and colon polypectomy) without significant bleeding.

### Conclusions

There are significant differences of opinion and practice patterns between Eastern and Western endoscopists based on significant differences on embolism, bleeding, and drug metabolism. The ratio of cerebral infarction to cardiovascular thromboembolism is different, and the recommended doses of antiplatelet and anticoagulant are smaller in the East due to different drug metabolism. The risk/benefit ratio of antiplatelets and anticoagulants may differ across population, and thus higher bleeding tendency due to higher concentration of drugs in Easterners should be considered.

Increased use of anticoagulants and antiplatelets will keep this as an argument not only to the GI endoscopists but also to the physicians prescribing these drugs. Both the concerns of endoscopists who want to stop medications to lessen the risk of hemorrhage, and the concerns of prescribing physicians who want to continue medications because of a fear of thromboembolic events, should always be considered based on patient’s individual characteristics such as a racial difference. Being aware of such wide variation between the East and West will be helpful in weighing the risk of bleeding and thromboembolism, when managing antiplatelets and anticoagulants for GI endoscopy.
